# Cox-2 Plays a Vital Role in the Impaired Anxiety Like Behavior in Colchicine Induced Rat Model of Alzheimer Disease

**DOI:** 10.1155/2016/1501527

**Published:** 2016-01-10

**Authors:** Susmita Sil, Tusharkanti Ghosh

**Affiliations:** Neurophysiology Laboratory, Department of Physiology, University College of Science and Technology, University of Calcutta, 92 Acharya Prafulla Chandra Road, Kolkata, West Bengal 700 009, India

## Abstract

The anxiety status is changed along with memory impairments in intracerebroventricular colchicine injected rat model of Alzheimer Disease (cAD) due to neurodegeneration, which has been indicated to be mediated by inflammation. Inducible cox-2, involved in inflammation, may have important role in the colchicine induced alteration of anxiety status. Therefore, the present study was designed to investigate the role of cox-2 on the anxiety behavior (response to novelty in an elevated open field space) of cAD by inhibiting it with three different doses (10, 20, and 30 mg) of etoricoxib (a cox-2 blocker) in two time points (14 and 21 days). The results showed anxiolytic behavior in cAD along with lower serum corticosterone level, both of which were recovered at all the doses of etoricoxib on day 21. On day 14 all of the anxiety parameters showed similar results to that of day 21 at high doses but not at 10 mg/kg body weight. Results indicate that the parameters of anxiety were dependent on neuronal circuitries that were probably sensitive to etoricoxib induced blocking of neurodegeneration. The present study showed that anxiolytic behavior in cADr is predominantly due to cox-2 mediated neuroinflammation induced neurodegeneration in the brain.

## 1. Introduction

Colchicine is a plant alkaloid and has neurotoxic properties [1, 2]. It is a microtubule depolymerizing agent and causes blocking of the axoplasmic flow in colchicine induced rat model of Alzheimer Disease (cAD) [1, 2]. If the axoplasmic flow is blocked, it affects the normal activity of the neurons resulting in its death [1, 2]. Intracerebroventricular injection of colchicine in the lateral ventricles of rat causes neurodegeneration in different brain areas including hippocampus and amygdala and as an outcome impairments of cognitive (e.g., memory) [3, 4] and noncognitive (e.g., anxiety behavior) functions occur [5]. Raghavendra et al. [6] have shown that cAD rats exhibit anxiogenic behavior in an elevated plus maze whereas Sil and Ghosh [5] have reported anxiolytic behavior in cAD rats in an elevated open field with a novel object in the centre along with lower level of serum corticosterone. The dissimilarity of anxiety behavior in the two studies may originate from the difference of the methods employed for the measurement of this behavior.

Sil et al. [4] have indicated that the colchicine induced neurodegeneration was mediated by neuroinflammation. These investigators have also reported that colchicine induced anxiolytic behavior was due to cyclooxygenase (cox) induced neuroinflammation mediated neurodegeneration [4, 5, 7]. Moreover, it was also reported that the anxiolytic behavior along with neurodegeneration was increased in cAD rats in 3 weeks of study compared to that of 2 weeks of study [5]. This time dependent increase in anxiolytic behavior was also prevented better by the administration of naproxen (general cox inhibitor) for 3 weeks of study compared to that of 2 weeks of study which could be corroborated with better protection of neuroinflammation and neurodegeneration by this inhibitor in longer study duration [5, 7]. Neuroinflammation of the brain in general and hippocampus in particular is found to be associated with two varieties of cox, that is, cox-1 and cox-2 [8]. It has been reported that cox-1 is constitutively expressed in brain and is specifically concentrated in pyramidal neurons of hippocampus and cerebral cortex [9]. Cox-2, on the other hand, is upregulated during inflammation in both the neurons and reactive microglia [10]. So blocking of cox-2 expression and activity by its specific blocker, etoricoxib, may efficiently protect inflammation and thereby may inhibit the impairment of anxiety behavior than that of nonspecific cox blockers (naproxen) in cAD rats. Kumar et al. [3] have reported that the blocking of cox-2 activity in cAD rats by valdecoxib (specific cox-2 inhibitor) resulted in better recovery of memory impairment, oxidative stress, and acetylcholine esterase activity compared to that of nonspecific cox blocker, naproxen, in a 25-day study indicating an important role of cox-2 on the colchicine induced memory impairments and stress parameters. Anxiety behavior is regulated by different central neural areas such as amygdala and hippocampus [5] which undergoes changes by the neurotoxic effect of colchicine. It is likely that anxiety may be influenced in cAD rats by the administration of cox-2 inhibitor as it can reduce oxidative stress and thereby the neurotoxic effects of colchicine on the central neural areas regulating anxiety may be prevented. If cox-2 inhibitor can influence the anxiolytic behavior in cAD rats by inhibiting colchicine induced neuroinflammation, the prolonged use of this cox-2 inhibitor may show better effect than that of short duration study. There is however no information in the literature regarding the effects of cox-2 blocker on the anxiety like behavior in cAD rats.

Therefore, the present study was designed to investigate the role of cox-2 on the different attributes of anxiety like behavior in icv colchicine injected AD rats by blocking its expression and activity by etoricoxib (a cox-2 blocker) in 14- and 21-day study.

## 2. Methods

### 2.1. Animals

144 healthy, adult male albino rats (Charles-Foster strain) weighing 200–250 g (6–8 weeks of age) were used in this study. Animals were housed individually in polypropylene animal cages with food pellet and water ad libitum. The animal room was maintained at the temperature of 25 ± 1°C with a 12 h light dark cycle (light 7 a.m. to 7 p.m.). According to the regulations set by institutional animal ethical committee, all adequate measures were taken to minimize the pain and discomfort in the rats.

### 2.2. Design of Experiments

144 rats were used in this study and were divided in the following way.

*14-Day Study*. 72 rats were divided into three division: control, sham (icv artificial CSF), and AD (icv colchicine injection). Each one was divided into 4 groups which contain 6 animals each: Group I (without any etoricoxib), Group II (10 mg/kg body wt. of etoricoxib), Group III (20 mg/kg body wt. of etoricoxib), and Group IV (30 mg/kg body wt. of etoricoxib). Therefore, 12 groups were used in this study. On 14th day after icv injection of colchicine/artificial CSF all the rats in each group were subjected to a 10 min anxiety test in an elevated open field in response to a novel object. After the end of the anxiety test rats were sacrificed (under ether anesthesia) between 11 and 11.30 a.m. and 1.5 mL blood was collected from the heart by a syringe and was kept for serum collection without any anticoagulant. With this serum corticosterone level was measured in different groups of rats. 

*21-Day Study*. 72 rats were divided similar to the 14-day study and thus 12 groups were used in this study. All the rats in the 21-day study were subjected to anxiety test after 21 days of icv colchicine/artificial CSF injection and then serum corticosterone levels were measured in these rats in the same manner like the 14-day study.

### 2.3. Drug Treatment

Etoricoxib (Ranbaxy, India) was dissolved in distilled water and it was administered orally through a gastric cannula attached to a 1 mL syringe. The daily dose of etoricoxib was divided equally into two parts given at 6-hour intervals each. Three doses of etoricoxib (10, 20, and 30 mg/kg body wt.) were given po to different groups of rats for 14 and 21 days each starting from 4 days prior to colchicine (icv) injection (for AD rats) and 4 days prior to vehicle (icv) injection (for sham operated rats). Control rats were also treated with etoricoxib for the same time period.

### 2.4. Preparation of Experimental Rat Model of Alzheimer's Disease by Colchicine

In the lateral ventricle of rat, 7.5 *μ*g of colchicine (SRL, India) dissolved in 2.5 *μ*L artificial CSF [3] was injected slowly for 5 minutes. The lateral ventricle of both sides of the brain was approached stereotaxically [11] (AP −0.6 mm from bregma, L ±1.5 mm, and V +2.8 mm below cortical surface) through a steel cannula (0.45 mm diameter) connected to a Hamilton syringe in anesthetized rats (Na-thiopentone, 50 mg/kg body wt. i.p.) and the method has been described in Sil et al. [7].

### 2.5. Measurement of Anxiety Behavior

The anxiety behavior was measured by the method described by Ennaceur et al. [12] in open field space. This method uses response of rats to novelty in an open space field. The apparatus and adopted method is briefly described below.

#### 2.5.1. Apparatus

The apparatus consists of a wooden platform (width 80 cm × length 80 cm × height 50 cm) elevated by 120 cm from the ground. The objects (width 8 cm × length 8 cm × height 13 cm) which were to be explored were in triplicate and were alternated between animals. They were made of white ceramic, a biologically neutral material. The objects were of heavy weight so that the animals could not move them around in the arena. These objects had never been associated with a reinforcer and are not known to have any ethological significance for the rats. The field of the open space was divided in outer area, inner area, and object area. The outer area was 15 cm wide from the edge of the field. The inner area was 10 cm × 10 cm wide located in the middle of the field. The object area is defined by a circle drawn in the centre, which is 2 cm larger than the bottom surface of the objects that was used in the experiment.

#### 2.5.2. Testing

The experiments were carried out in open space with an object in the centre. These required testing animals in single session on 14th and 21st day after colchicine or vehicle injection along with control rats for all the groups. In each session, all the rats in each group were tested for 10 min. The surface of the platform of the apparatus was cleaned with 90% ethanol after testing of each rat and left to dry before the introduction of the next rat to minimize the effects of lingering olfactory cues. Rats were released into an open space from any part of the outer area (randomly predefined) facing away from the inner area. They were released for 10 min to explore the area of the open space. Rats were observed on a screen monitor connected to a video camera suspended above the test arena. The latency of first entry to the inner area, frequency of entry to the inner area, total time spent in the inner area, total time spent in the outer area, latency of first approach to the object area, and frequency of approach to the object area were noted in each session.

### 2.6. Blood Collection

Blood was collected (1.5 mL, between 11:00 a.m. and 11:30 a.m.) from the heart of anesthetized rat (Diethyl Ether, Kabra Drugs, India) by a syringe and was kept for serum collection without any anticoagulant. With this serum corticosterone level was measured in different groups of rats in 14- and 21-day study.

### 2.7. Corticosterone Level

Serum corticosterone (CORT) concentration was determined by radioimmunoassay using a commercially available kit (^125^I Rat Corticosterone (MP Biomedicals, LIC, Diagnostics Division, Ohio)) and gamma counter. The antisera used for the assay were highly specific for the Rat Corticosterone and it had 1.58% cross-reactivity with 11-deoxycorticosterone. The assay sensitivity is approximately 10 ng/mL and intra-assay-interassay coefficient variation was less than 10%. Quality control serum was used for the assay and all the experimental samples were run in duplicate.

### 2.8. Statistical Analysis

Data are expressed as mean ± SEM. One way ANOVA was employed to compare the data of the control, sham operated, and AD experimental groups followed by Tukey's multiple comparison test using the Statistical Package for Social science Software (SPSS software: 20.0.0, USA).

## 3. Results

### 3.1. Parameters of Anxiety

#### 3.1.1. Latency of First Entry to the Inner Area

The latency of first entry to the inner area was significantly increased [*F*(23, 120) = 4.981, (*p* < 0.001)] in cAD rats on both the days 14 and 21 compared to that of respective control (C) and sham operated (S) rats. After administration of etoricoxib in cAD rats at the doses of 10 mg, 20 mg, and 30 mg/kg body weight, the latency of first entry to the inner area was significantly lower (*p* < 0.001) compared to cAD rats on both the days 14 and 21. The latency of first entry to the inner area was not significantly changed among the eight groups of control and sham rats on both the days 14 and 21 (Table 1).

#### 3.1.2. Frequency of Entry to the Inner Area

**Figure 1:**
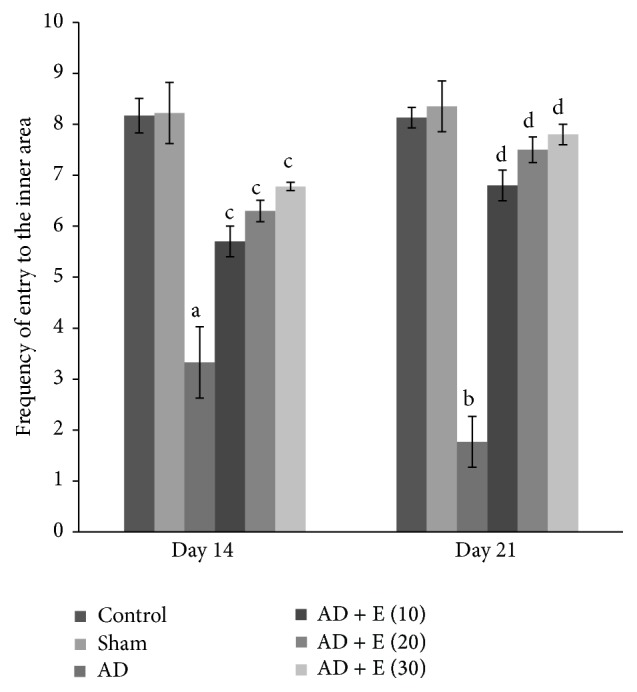
The frequency of entry to the inner area in an elevated open space with a novel object (for anxiety status) of different experimental groups of rats. ^a^cAD versus control/sham operated rats at day 14 (*p* < 0.01), ^b^cAD versus control/sham operated rats at day 21 (*p* < 0.001), ^c^cAD versus etoricoxib treated cAD at all the doses at day 14 (*p* < 0.001), and ^d^cAD versus etoricoxib treated cAD at all the doses at day 21 (*p* < 0.001). AD: intracerebroventricular colchicine injected Alzheimer Disease rats, AD + E (10): AD rats treated with 10 mg/kg body weight of etoricoxib, AD + E (20): AD rats treated with 20 mg/kg body weight of etoricoxib, and AD + E (30): AD rats treated with 30 mg/kg body weight of etoricoxib. Values are expressed in mean ± SEM (*n* = 6 in each group).

Frequency of entry to the inner area significantly decreased [*F*(23,120) = 4.264] in cAD rats on days 14 (*p* < 0.05) and 21 (*p* < 0.001) compared to that of respective control (C) and sham operated (S) rats. After administration of etoricoxib in cAD rats at the doses of 10, 20, and 30 mg/kg body weight, frequency of entry to the inner area was significantly higher (*p* < 0.001) compared to cAD rats on both the days 14 and 21. The frequency of entry to the inner area was not significantly changed among the eight groups of control and sham rats on both the days 14 and 21 (Figure 1).

#### 3.1.3. Total Time Spent in the Inner Area

**Figure 2:**
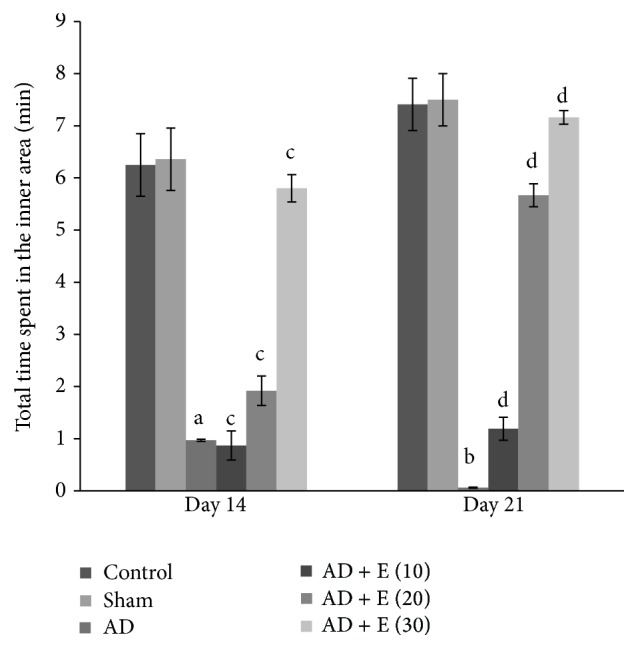
The total time spent in the inner area in an elevated open space with a novel object (for anxiety status) of different experimental groups of rats. ^a^cAD versus control/sham operated rats at day 14 (*p* < 0.001), ^b^cAD versus control/sham operated rats at day 21 (*p* < 0.001), ^c^cAD versus etoricoxib treated cAD at all the doses at day 14 (*p* < 0.001), and ^d^cAD versus etoricoxib treated cAD at all the doses at day 21 (*p* < 0.001). Abbreviations are the same as in Figure 1. Values are expressed in mean ± SEM (*n* = 6 in each group).

The total time spent in the inner area significantly decreased [*F*(23,120) = 6.290, (*p* < 0.001)] in cAD rats on both the days 14 and 21 compared to that of respective control (C) and sham operated (S) rats. After administration of etoricoxib in cAD rats at the doses of 10, 20, and 30 mg/kg body weight, total time spent in the inner area was significantly higher (*p* < 0.001) compared to cAD rats on both the days 14 and 21. The total time spent in the inner area was not significantly changed among the eight groups of control and sham rats on both the days 14 and 21 (Figure 2).

#### 3.1.4. Total Time Spent in the Outer Area

**Figure 3:**
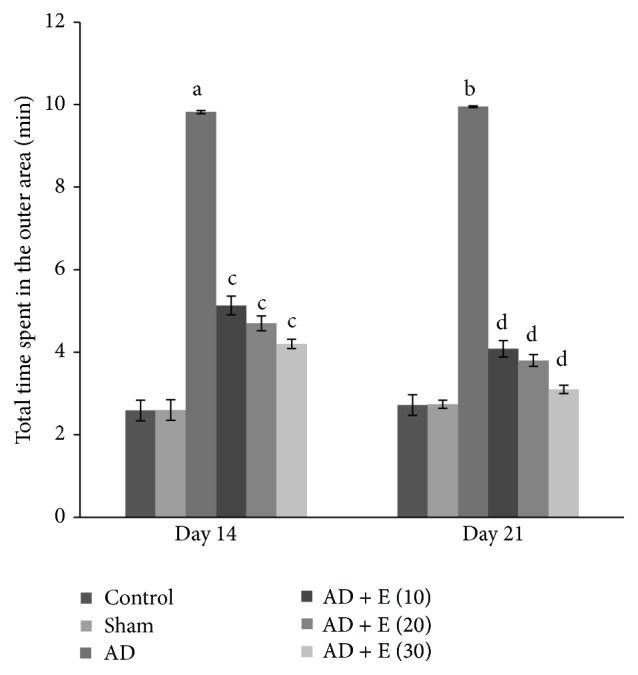
The total time spent in the outer area in an elevated open space with a novel object (for anxiety status) of different experimental groups of rats. ^a^cAD versus control/sham operated rats at day 14 (*p* < 0.001), ^b^cAD versus control/sham operated rats at day 21 (*p* < 0.001), ^c^cAD versus etoricoxib treated cAD at all the doses at day 14 (*p* < 0.001), and ^d^cAD versus etoricoxib treated cAD at all the doses at day 21 (*p* < 0.001). Abbreviations are the same as in Figure 1. Values are expressed in mean ± SEM (*n* = 6 in each group).

The total time spent in the outer area significantly increased [*F*(23,120) = 95.868, (*p* < 0.001)] in cAD rats on both the days 14 and 21 compared to that of respective control (C) and sham operated (S) rats. After administration of etoricoxib in cAD rats at the doses of 10, 20, and 30 mg/kg body weight, total time spent in the outer area was significantly lower (*p* < 0.001) compared to cAD rats on both the days 14 and 21. The total time spent in the outer area was not significantly changed among the eight groups of control and sham rats on both the days 14 and 21 (Figure 3).

#### 3.1.5. Latency of First Approach to the Object Area

**Figure 4:**
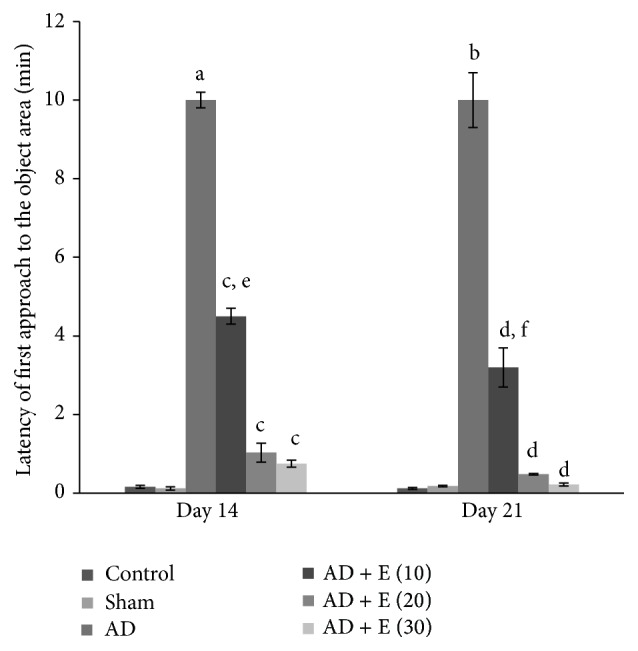
The latency of first approach to the object area in an elevated open space with a novel object (for anxiety status) of different experimental groups of rats. ^a^cAD versus control/sham operated rats at day 14 (*p* < 0.001), ^b^cAD versus control/sham operated rats at day 21 (*p* < 0.001), ^c^cAD versus etoricoxib treated cAD at all the doses at day 14 (*p* < 0.001), ^d^cAD versus etoricoxib treated cAD at all the doses at day 21 (*p* < 0.001), ^e^10 mg/kg body weight etoricoxib administered cAD versus 20, 30 mg/kg body weight etoricoxib administered cAD at day 14 (*p* < 0.001), and ^f^10 mg/kg body weight etoricoxib administered cAD versus 20, 30 mg/kg body weight etoricoxib administered cAD at day 21 (*p* < 0.001). Abbreviations are the same as in Figure 1. Values are expressed in mean ± SEM (*n* = 6 in each group).

The latency of first approach to the object area significantly increased [*F*(23,120) = 34.635, (*p* < 0.001)] in cAD rats on both the days 14 and 21 compared to that of respective control (C) and sham operated (S) rats. After administration of etoricoxib in cAD rats at the doses of 10, 20, and 30 mg/kg body weight, latency of first approach to the object area was significantly lower (*p* < 0.001) compared to cAD rats on both the days 14 and 21. The increase in latency of first approach to the object area of cAD rats after administration of different doses of etoricoxib showed a graded effect and there was significant difference between cAD rats treated with 10 mg and 20/30 mg/kg body weight of etoricoxib (*p* < 0.001) on both the days 14 and 21. Latency of first approach to the object area was not significantly changed among the eight groups of control and sham rats on both the days 14 and 21 (Figure 4).

#### 3.1.6. Frequency of Approach to the Object Area

**Figure 5:**
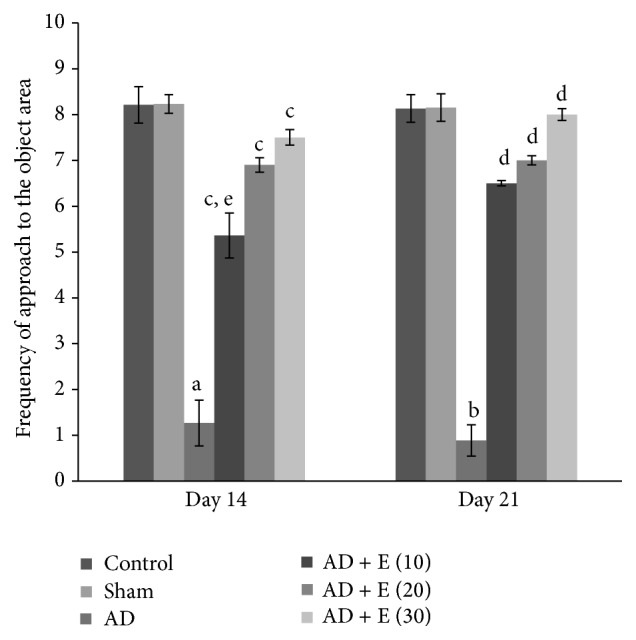
The frequency of approach to the object area in an elevated open space with a novel object (for anxiety status) of different experimental groups of rats. ^a^cAD versus control/sham operated rats at day 14 (*p* < 0.001).  ^b^cAD versus control/sham operated rats at day 21 (*p* < 0.001). ^c^cAD versus etoricoxib treated cAD at all the doses at day 14 (*p* < 0.001), ^d^cAD versus etoricoxib treated cAD at all the doses at day 21 (*p* < 0.001), and ^e^10 mg/kg body weight etoricoxib administered cAD versus 30 mg/kg body weight etoricoxib administered cAD at day 14 (*p* < 0.001). Abbreviations are the same as in Figure 1. Values are expressed in mean ± SEM (*n* = 6 in each group).

The frequency of approach to the object area significantly decreased [*F*(23,120) = 34.257, (*p* < 0.001)] in cAD rats on both the days 14 and 21 compared to that of respective control (C) and sham operated (S) rats. After administration of etoricoxib incAD rats at the doses of 10, 20, and 30 mg/kg body weight, frequency of approach to the object area did not decrease and it was significantly higher (*p* < 0.001) compared to cAD rats on both the days 14 and 21. The increase in frequency of approach to the object area of cAD rats after administration of different doses of etoricoxib showed a graded effect and there was significant difference between cAD rats treated with 10 mg and 30 mg/kg body weight of etoricoxib (*p* < 0.05) on day 14. The frequency of approach to the object area was not significantly changed among the eight groups of control and sham rats on both the days 14 and 21 (Figure 5).

#### 3.1.7. Serum Corticosterone Level (ng/mL)

**Figure 6:**
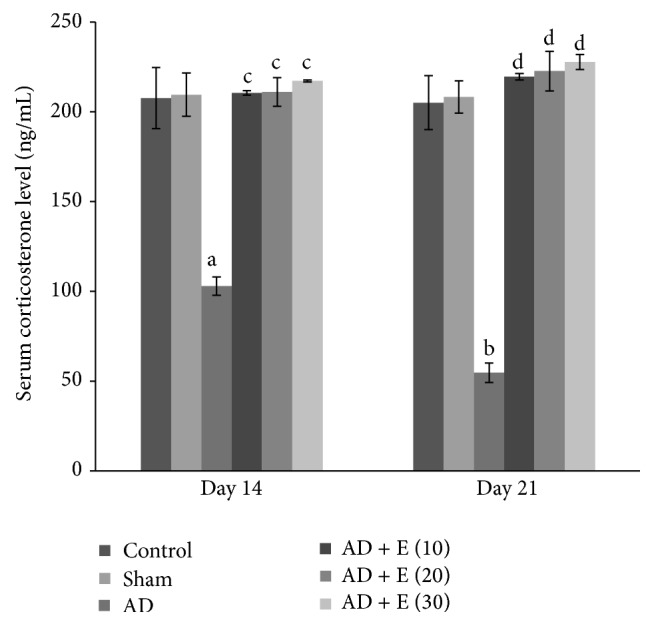
Serum corticosterone level in different experimental groups of rats. ^a^cAD versus control/sham operated rats at day 14 (*p* < 0.001). ^b^cAD versus control/sham operated rats at day 21 (*p* < 0.001). ^c^cAD versus etoricoxib treated cAD at the doses of 10, 20, and 30 mg/kg body weight at day 14 (*p* < 0.001). ^d^cAD versus etoricoxib treated cAD at the doses of 10, 20, and 30 mg/kg body weight at day 21 (*p* < 0.001). Abbreviations are the same as in Figure 1. Values are expressed in mean ± SEM (*n* = 6 in each group).

The serum corticosterone level was significantly decreased [*F*(23,120) = 6.236, (*p* < 0.001)] in cAD rats on both the days 14 and 21 compared to that of respective control (C) and sham operated (S) rats. After administration of etoricoxib in cAD rats at the doses of 10, 20, and 30 mg/kg body weight, serum corticosterone level reached the control level and was significantly higher (*p* < 0.001) compared to cAD rats on both the days 14 and 21. The serum corticosterone level was not significantly changed among the eight groups of control and sham rats on both the days 14 and 21 (Figure 6).

## 4. Discussion

Though any animal model may not always exhibit all the characteristic features of a disease but some salient features of the disease are found in the animal models [13]. The neuropathology in cAD rats may not be exactly similar to AD as colchicine induces nonspecific neurodegeneration. Intracerebroventricular colchicine injected model of AD rats produced neurodegeneration and amyloid plaques in different parts of the brain which had similarity with the pattern of AD pathology [4]. The impairment of memory in colchicine induced AD rats also showed similarity with AD patients [14–24]. This model has been considered as sporadic model of AD by many investigators and has been used as a model for the development of new drugs in AD [14–24]. Therefore, icv colchicine injected model is relevant for investigating different behavioral and neuropathological features of AD.

Anxiety along with dementia in AD patients has been reported for a long time. Though memory impairment is exhibited in most of the animal models of AD, the anxiety behavior is variable to some extent. The anxiety behavior depends not only on the animal models of AD but also on the methods of investigation [12]. The anxiety status in different transgenic mice, as reported by several investigators, is inconsistent and sometimes contradictory when observed in similar methods [25–27]. In elevated plus maze (EPM) anxiogenic behavior was observed in Tg 2576 mice, but anxiolytic behavior was noted in other types of transgenic mice (mutant PS1 and PS2 genes) and still in transgenic mice overexpressing APP, anxiety status was not changed [25–27]. The anxiety behavior was found to be different in same animal models of AD by different investigators using same method of measurement [28–30]. The anxiety status in cAD rats was also not similar in two studies [5, 6]. The results of the present study show that the latency of entry, frequency of entry and total time spent in the inner area, latency of first approach, and frequency of approach to object area have increased and the total time spent in the outer area has decreased in cAD rats on days 14 and 21. These results indicate an anxiolytic behavior in cAD rats of the present study. Sil and Ghosh [5] have reported previously that the anxiety like behavior was reduced in cAD rats in elevated open field with a novel object, while Raghavendra et al. [6] have reported opposite anxiety status, that is, anxiogenic behavior in the similarly prepared cAD rats in an elevated plus maze. Though the animal models were similarly prepared in these two later studies, the methods of behavioral assessment were different in these studies. The reduced anxiety like behavior in cAD rats was not possibly due to impairment of locomotor activity since locomotor activity was not affected in cAD rats [13]. Anxiolytic behavior in experimental animal model of AD is difficult to explain considering the anxiogenic symptoms found in AD patients. However, Yuk et al. [30] concluded from the anxiolytic behavior in PS2 transgenic mice of AD that rodent behavior could not be extrapolated to human patients and opined that the anxiety might be reduced in human patients with progression of AD. The anxiety like behavior in cAD rats was changed after administration of etoricoxib on days 14 and 21. Based on the statistical analysis of the results it was found that the anxiety in cAD rats after treatment with three different doses of Etoricoxib (po) was reduced completely, or partially to the level of control as indicated by some of the test parameters used in the present study (latency of first entry to inner and object area, frequency of entry to inner and object area, and total time spent in the inner and outer area). Some parameters of anxiety (frequency of entry to object area and total time spent in the inner area) were reduced partially to the level of control rats at the dose of 10 mg/kg body weight of etoricoxib in cAD rats, while these parameters were reduced completely to the level of control at the doses of 20 and 30 mg on day 14. On day 21 all the observed parameters were reduced completely to the level of control at all the doses of etoricoxib. Therefore the results indicate that etoricoxib administration at the lowest dose (10 mg/kg body weight) is able to inhibit the changes of most of the parameters of anxiety in cAD rats.

It appears from these results that the parameters of anxiety (e.g., latency of first entry to inner area, frequency of entry to inner area, total time spent in the inner area, and latency of first approach to the object area) which were reduced completely to the level of control at the dose of 10 mg/kg body weight of etoricoxib in cAD rats on day 14 were dependent on the neuronal circuitry that were probably more sensitive to etoricoxib induced blocking of neurodegeneration than that of the other parameters of anxiety (total time spent in the outer area and frequency of approach to the object area) which did not reduce to the level of control at the dose of 10 mg/kg body weight of etoricoxib in cAD rats at the same time point. The anxiolytic behavior in the cAD rats may be the sum total effect of the impaired function of different neural areas which were affected by colchicine induced neurodegeneration. The protection from neurodegeneration of these brain areas after administration of etoricoxib may be the cause behind recovery from anxiolytic behavior observed in the present study. Etoricoxib can cross the blood brain barrier [31] and thereby may inhibit colchicine induced neurodegeneration. Nonspecific cox inhibitor, naproxen, can also cross the blood brain barrier [32] and may inhibit colchicine induced neurodegeneration like that of etoricoxib [7]. But etoricoxib appears to be more effective in this regard compared to naproxen (data reported earlier) [5]. It was found in the comparative analysis of the data of these two drugs (data not shown) that all of the test parameters were recovered to control at the dose of 10 mg/kg body weight of etoricoxib on day 21 but naproxen showed similar results at 20 mg/kg body weight at same time point. The better protective effect of etoricoxib on anxiolytic behavior in cAD rats than that of naproxen also may be supported by the observation that the total time spent in the outer area (parameter of anxiety) in cAD rats regains the level of control by administration of etoricoxib at the dose of 20 mg/kg body weight on day 14, whereas it did not regain the control value with naproxen at the same dose and time point. The better protection of anxiolytic behavior by etoricoxib in comparison to naproxen may be due to the dual inhibitory action of etoricoxib on the expression and activity of cox-2 [33, 34], while naproxen can only inhibit both the cox-1 and cox-2 activity but not their expression [35, 36]. Since inducible cox-2 expression and activity were increased during neuroinflammation [37], the blocking of this enzyme expression and activity by etoricoxib [33, 34] in the present study probably inhibited neuroinflammation, and as a result neurodegeneration was also inhibited in these rats. Therefore, the role of cox-2 on the colchicine induced anxiolytic behavior was emphasized from this study.

The lower serum corticosterone level was observed in the cAD rats and the administration of etoricoxib in these animals resulted in complete recovery of corticosterone levels at the lowest observed dose (10 mg/kg body wt.) on both the days 14 and 21 and continued to remain so at all the other observed doses in this study. This recovery of corticosterone in etoricoxib treated cAD rats can be corroborated with the recovery of anxiolytic behavior in these rats. The serum corticosterone level in cAD rats may be related with the anxiety status of the animals. It has been reported that the subcutaneous injection of corticosterone in rats [38] or intracerebroventricular injection of corticosterone releasing factor in rats [39] resulted in anxiogenic behavior in these animals. It was also found that the anxiolytic behavior in rats was accompanied by lower serum corticosterone level [40]. The low serum corticosterone level of the present study is probably linked with the anxiolytic behavior of the cAD rats in addition to the neurodegeneration of the brain areas regulating anxiety. The hypothalamo-hypophysial-adrenal (HPA) axis may be depressed by the low CRH secretion in cAD rats due to the colchicine induced damage of the neurons that are involved in the regulation of CRH secretion from hypothalamus and inhibition of this neuronal damage by etoricoxib may help to recover the normal activity of the HPA axis. The present study showed that the anxiolytic behavior in cAD rats originated due to colchicine induced increased activity of the cox-2 which played a key role in the inflammation and later degeneration in the brain. The participation of cox-1 on neurodegeneration, though negligible due to its noninducible character, has not been assessed in this study.

## 5. Conclusion

The present study showed that anxiolytic behavior in cAD rats is predominantly due to cox-2 mediated neuroinflammation induced neurodegeneration in the brain.

## Figures and Tables

**Table 1:** The latency of first entry to the inner area in an elevated open space with a novel object (for anxiety status) of different experimental groups of rats.

Groups	Day 14	Day 21
Control	0.09 ± 0.34	0.02 ± 0.29
Control + E (10 mg/kg)	0.07 ± 0.30	0.06 ± 0.27
Control + E (20 mg/kg)	0.06 ± 0.20	0.08 ± 0.22
Control + E (30 mg/kg)	0.07 ± 0.34	0.06 ± 0.28
Sham	0.08 ± 0.36	0.09 ± 0.30
Sham + E (10 mg/kg)	0.08 ± 0.34	0.04 ± 0.28
Sham + E (20 mg/kg)	0.07 ± 0.32	0.08 ± 0.31
Sham + E (30 mg/kg)	0.09 ± 0.34	0.06 ± 0.29
AD	3.73 ± 2.00^a^	6.11 ± 1.47^b^
AD + E (10 mg/kg)	0.98 ± 0.36^c^	0.26 ± 0.02^d^
AD + E (20 mg/kg)	0.72 ± 0.03^c^	0.13 ± 0.01^d^
AD + E (30 mg/kg)	0.33 ± 0.01^c^	0.11 ± 0.14^d^

^a^cAD versus control/sham operated rats at day 14 (*p* < 0.001), ^b^cAD versus control/sham operated rats at day 21 (*p* < 0.001), ^c^cAD versus etoricoxib treated cAD at all the doses at day 14 (*p* < 0.001), and ^d^cAD versus etoricoxib treated cAD at all the doses at day 21 (*p* < 0.001). Control + E (10 mg/kg): control rats treated with 10 mg/kg body weight of etoricoxib, control + E (20 mg/kg): control rats treated with 20 mg/kg body weight of etoricoxib, control + E (30 mg/kg): control rats treated with 30 mg/kg body weight of etoricoxib, sham + E (10 mg/kg): sham operated rats treated with 10 mg/kg body weight of etoricoxib, sham + E (20 mg/kg): sham operated rats treated with 20 mg/kg body weight of etoricoxib, sham + E (30 mg/kg): sham operated rats treated with 30 mg/kg body weight of etoricoxib, AD: intracerebroventricular colchicine injected Alzheimer disease rats, AD + E (10 mg/kg): AD rats treated with 10 mg/kg body weight of etoricoxib, AD + E (20 mg/kg): AD rats treated with 20 mg/kg body weight of etoricoxib, and AD + E (30 mg/kg): AD rats treated with 30 mg/kg body weight of etoricoxib. Values are expressed in mean ± SEM (*n* = 6 in each group).
